# Alpha rhythm collapse predicts iso-electric suppressions during anesthesia

**DOI:** 10.1038/s42003-019-0575-3

**Published:** 2019-09-02

**Authors:** Jérôme Cartailler, Pierre Parutto, Cyril Touchard, Fabrice Vallée, David Holcman

**Affiliations:** 1 Group of Data Modeling, Computational Biology and Predictive Medicine, Institut de Biologie de l’École Normale Supérieure (IBENS); École Normale Supérieure CNRS/INSERM, Université PSL, Paris, France; 2 Department of Anesthesiology and Critical Care, St-Louis- Lariboisière-Fernand Widal University Hospitals, Assistance Publique - Hôpitaux de Paris (AP-HP), Paris, France; 3 Department of Biochemistry and DAMPT, University of Cambridge and Churchill College, Cambridge, UK

**Keywords:** Translational research, Learning algorithms, Risk factors

## Abstract

Could an overly deep sedation be anticipated from ElectroEncephaloGram (EEG) patterns? We report here motifs hidden in the EEG signal that predict the appearance of Iso-Electric Suppressions (IES), observed during epileptic encephalopathies, drug intoxications, comatose, brain death or during anesthetic over-dosage that are considered to be detrimental. To show that IES occurrences can be predicted from EEG traces dynamics, we focus on transient suppression of the alpha rhythm (8–14 Hz) recorded for 80 patients, that had a Propofol target controlled infusion of 5 μg/ml during a general anesthesia. We found that the first time of appearance as well as changes in duration of these Alpha-Suppressions (*α*S) are two parameters that anticipate the appearance of IES. Using machine learning, we predicted IES appearance from the first 10 min of EEG (AUC of 0.93). To conclude, transient motifs in the alpha rhythm predict IES during anesthesia and can be used to identify patients, with higher risks of post-operative complications.

## Abstract (web summary)

Jerome Cartailler, Pierre Parutto et al. identify motifs in electroencephalogram (EEG) recordings that predict brain patterns seen in excessive anesthesia. The EEG motifs were detected within the first few minutes of general anesthesia, suggesting they could be used to monitor patients during procedures and avoid over-sedation.

## Introduction

Various frequency bands can be extracted from the electroencephalogram (EEG), but also transient patterns such as iso-electrical suppressions (IES) that consist of periods of iso-electric (flat) activity lasting from few seconds to several minutes^1^. These suppressions compose the quiescent part of the Burst-Suppression (BS) pattern and appear in several pathological conditions such as epileptic encephalopathies^2^, drug intoxications^3^, comatose^4^ or brain death^5^. They are also associated with post-operative sleep disorder^6^, the emergence of delirium^7,8^, and an increased mortality in sedated critically ill patients^9^. IES can also appear when the concentrations of anesthetics increases^10^. Avoiding anesthetic drug-related IES is a recommendation during anesthesia, yet there are no robust methods to anticipate and prevent IES^11^.

EEG monitoring is currently used to track loss of consciousness in real time^12^ and to evaluate the depth of anesthesia^13^ using sophisticated algorithms that can also detect the presence of IES^14^. However, no causal relation between rhythms has been documented today to predict the appearance of IES. In a propofol-induced general anesthesia (GA) the main rhythms consist of the alpha-rhythm ([8–14] Hz) and a delta-band ([0.5–5] Hz) activity^15^, unless the rhythm breaks and IES develop. It is unclear whether IES appear as the result of drug-induced boosting of cortical post-synaptic inhibitory currents or a decreased cerebral metabolic rate of oxygen (CMRO)^10,16,17^, which could lead to a damaging cerebral hypoxia.

To predict how a collapse of the rhythmic alpha-delta activity leads to IES transient periods, we developed here a methodology based on new EEG motifs that consist in partial and transient suppression of the alpha-rhythm (*α*S). We show that these changes (in duration or amplitude) can reliably predict IES occurrences during propofol-induced GA, which was suggested in^12,18–20^. We developed an algorithm based on wavelets and local signal amplitude enhancement analysis to detect periods of *αS*. The manuscript is divided into three parts: first, we present the relation between *α*S and IES relative proportions and occurrences in the EEG signal, demonstrating that *α*S precede IES; in the second part we compare *α*S properties time course for patients that developed IES versus those that did not. In the last part, we use classification machine learning algorithms to demonstrate that the transient statistical properties of the *α* band predict IES occurrences with a remarkably good score and thus reveal patients at risk during the first 10 min of GA. We conclude from the present study that transient motifs in the alpha rhythm predict IES during anesthesia and offer a perspective to detect cerebral fragility and anticipate post-operative complications.

## Results

### Alpha-suppressions (*α*S) anticipate IES during anesthesia

**Fig. 1:**
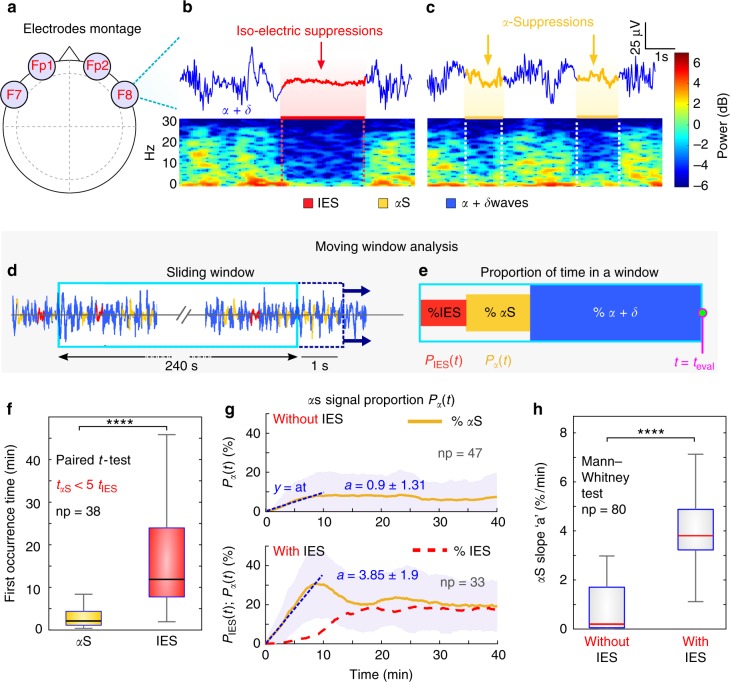
*α*S and iso-electric suppressions in EEG signal during GA. **a** Schematic representation of four frontal electrodes (F7, Fp1, Fp2, F8) to record EEG. **b**, **c** EEG signal and its associated spectrogram showing Iso-Electrical Suppressions (IES) (**b**, upper panel, red) and frequency loss in the alpha band (8–14 Hz), called *α*-Suppressions (**c**, upper panel, yellow). The hypnotic EEG signal (blue) contains both *α* + *δ* waves. **d** Schematic representation of the sliding window of width 240 s, translated every 1 s. **e** Proportion of *α*S (yellow), IES (red) and *α* + *δ* waves (blue) contained in a sliding window from (**d**). **f** Distribution of first occurrence times of *α*S (yellow) and IES (red) for *np* = 38 patients having both events (evaluated during 180 min). **g** Time course of *α*S (yellow) (resp. IES (red)) proportions *P*_*α*_(*t*) (resp. *P*_*IES*_) computed with the sliding widow (see **d**, **e**), for two populations: without (*np* = 47) and with (*np* = 33) IES. The slope (dashed blue) at time zero is equal to *a* = 0.9 ± 1.31 and 3.85 ± 1.9 for patients without and with IES respectively. **h** Distribution of the slope *a* defined in (**g**), for *np* = 80

We used the EEG signal monitored during GA with four frontal electrodes Fp1, Fp2, F7 and F8 (Fig. 1a) to detect *α*-Suppressions (*α*S) and iso-electrical suppressions (IES) (Fig. 1b, c), using the segmentation procedure described in’Methods’. We applied this procedure to the EEG of 80 patients and distinguished two populations characterized by a low (<10 s) (*np* = 47) and a more important (≥10 s) (*np* = 33) fraction of IES during the first 35 min of GA (Methods).

We computed for the two populations the percentage of time spent in *α*S, IES and *α* + *δ* (hypnosis) using a sliding window procedure (Fig. 1d, e). We performed this analysis during two phases of GA: induction (10 min) and maintenance <180 min (Table 1). We found that during induction (resp. maintenance) patients with IES had 1.9 ± 2.9%, 11.6 ± 8.1% and 86.4 ± 9.3% (resp. 10.5 ± 8.7%, 19.4 ± 9.2% and 70.1 ± 10.5%) of IES, *α*S and *α* + *δ* respectively, where the notation *α* + *δ* indicates that both rhythms are present in the EEG. Comparatively, patients without IES had 2.7 ± 4.0% and 97.3 ± 4.1% (resp. 6.7 ± 8.8% and 93.0 ± 9.3%) of *α*S and *α* + *δ* respectively. The group without IES the first 35 min had 0.3 ± 0.7% IES during the maintenance phase. Additionally, we found that 43% (n = 18) of patients with IES, had their first IES after the induction period, showing that occurrence of IES during induction is not a predictive marker. At this stage we concluded that patients without IES or few *α*S during the first 10 min shown almost no IES during the maintenance phase. In contrast, patients that already had more IES or *α*S during the induction phase had a larger fraction of IES (35 times more) during maintenance.

**Table 1:** Propofol TCIentage of *α*S, IES and *α* + *δ* computed during 10 min (induction) and 180 min of the maintenance phase for: all patients, patients with and without IES in the first 35 min

Events (%)		*α* + *δ*	*α*S	IES
Patient with IES (np = 33)	Ind. (10 min)	86.4	11.6	1.9
	Maint. (180 min)	70.1	19.4	10.5
Without IES (np = 47)	Ind.	97.3	2.7	0
	Maint.	93.0	6.7	0.3
All patients (np = 80)	Ind.	92.8	6.4	0.8
	Maint.	83.4	11.9	4.5

We then restricted our analysis to the first 35 min where no NMDA antagonizing drugs, such as ketamine, have been used. During that period of time, we asked whether *α*S could precede IES, and therefore be used as predictive factors. To address this question, we restricted ourselves to a subpopulation (np = 38) for which the EEG contained both *α*S and IES events, then we estimated the time *t*_*αS*_ (resp. *t*_IES_) of the first occurrence of an *α*S (resp. IES) event (“Methods”). To define these occurrence times, we computed the fraction *P*_*α*_ (resp. *P*_IES_) of *α*S (resp. IES) time course, using a 240*s* width sliding window (Fig. 1d, e), ultimately the first occurrence time corresponded to the first time proportions reached 5%. A statistical analysis showed that the time *t*_*αS*_ of *α*S (Median; IQR) *t*_*αS*_ = 2.11;3.28 min preceded the time *t*_IES_ = 11.88;16.23 min of first IES appearance (Fig. 1f): in averaged *t*_IES_ was five times larger than *t*_*αS*_ (paired *t*-test, *P*-value = 0.0001, one-tailed).

We then explored if the statistics associated with *α*S could differentiate between the populations containing or not IES. To address this question we computed the *α*S proportion *P*_*α*_(*t*) (Fig. 1g, yellow curves) for each subpopulation (individual traces in Supplementary Fig. 1). We found two different behaviors characterized by their initial increase and steady state. To compare the two populations, we estimated the initial slope *a* of the proportion *P*_*α*_ of *α*S. We obtained a slope *a* = 0.90 ± 1.31 %min^−1^ (*R*^2^ = 0.96) for patients with no IES, compared to *a* = 3.85 ± 1.90 %min^−1^ (*R*^2^ = 0.97) for patients with IES. For the group without IES, *P*_*α*_ plateaued at the value 7.85%, while for the other one, *P*_*α*_ showed underdamped oscillations, converging to a plateau value 18.5% (Fig. 1g). Finally, to confirm that the slope *a*, computed during the induction phase, was significantly different between patients from the two groups, we plotted the distribution estimated over a population of np = 80 patients, and we found a clear separation between the two populations (Fig. 1h) with (Median; IQR) *a*_noIES_ = 3.80;1.66 % min^−1^ for patients without IES and *a*_IES_ = 0.20;1.66 % min^−1^ for patients with IES (Mann–Whitney test, *P*-value = 0.0001 one-tailed, Fig. 1h). These results show that, in the present dataset, *α*S events estimated during the first 10 min, could be used to anticipate the occurrence of IES.

### *α*S events separate patients with an without IES

**Fig. 2:**
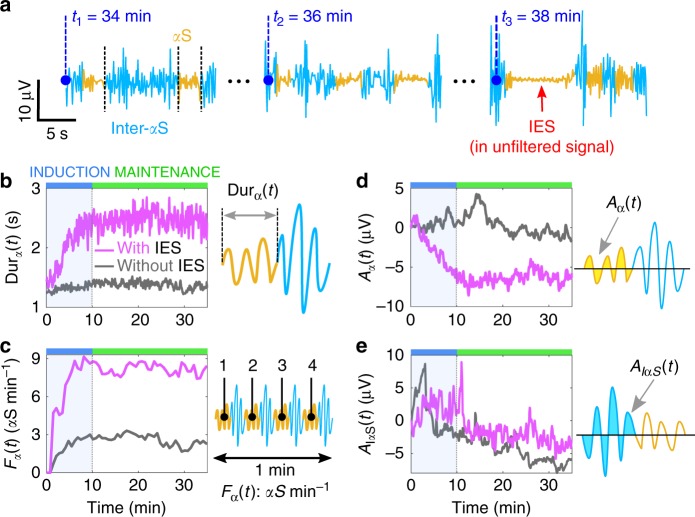
*α*S characteristics time-course for patients with and without IES. **a** Filtered signal *S*_*α*_(*t*) (see’Methods’) segmented in *α*S (yellow) and inter-*α*S (blue) for three different time points: *t*_1_ = 34, *t*_2_ = 36 and *t*_3_ = 38 min (electrode: Fp2). **b** *α*S duration averaged over np = 47 and np = 33 for patients with (magenta) and without (gray) IES, respectively. **c** *α*S occurrence frequency *F*_*α*_. **d**, **e** Time-series of averaged amplitude computed for each *α*S (**d**) and each inter-*α*S (**e**) (see (**a**) and “Method section”)

To differentiate the EEG signal of patients with and without IES, we decided to look for three statistical variables extracted from the *S*_*α*_(*t*) signal (Fig. 2a). We subsequently segmented the signal *S*_*α*_(*t*) into *α*S (yellow) and the rest in blue that we call Inter-*α*S (I*α*S) corresponding to regions between two consecutive *α*S. Note that I*α*S includes both bursting periods as well as continuous alpha activity in the filtered signal *S*_*α*_(*t*) (three examples are shown in Fig. 2a). In Fig. 2a, the *α*S with the smaller amplitude and the longest duration corresponded to an IES in the unfiltered signal.

We first estimated the duration Dur_*α*_ of *α*S events averaged over each group (Fig. 2b) and found that during the induction phase, *α*S duration increased from 1 to 2.5 s for the IES group, in contrast for the second group it was almost constant with mean 〈Dur_*α*_〉 = 1.24 s. To further characterize the induction phase, we computed the mean behavior of Dur_*α*_ by estimating for each group the slope *γ* of the fitted curves *y*(*t*) = *γt* + *b*, where *b* is a constant. We found *γ* = 0.02 s min^−1^ (resp. *γ* = 0.16 s min^−1^) for the group without IES (gray) (resp. with IES (magenta)). At this stage, these results suggest that the *α*S duration has a tendency to increase for the IES group only. These properties were confirmed during the first 35 min of the maintenance phase where the duration Dur_*α*_ for the IES group plateaued, while the group without IES remained at a baseline value of 1.3 s.

Second, we focused on changes in the occurrence of *α*S. For that purpose, we computed *α*S occurrence frequency *F*_*α*_ defined as the number of *α*S events per minutes (“Methods”). Computing *F*_*α*_ (Fig. 2c) showed a clear distinction between each group: although *F*_*α*_ is in both case increasing, it plateaus at 8.3 min^−1^ for IES group and 2.8 min^−1^ for the second group. Indeed, we fitted *F*_*α*_ with a single exponential *y*(*t*) = *F*_max_(1 − exp(−*t*/*τ*)), where *F*_max_ and *τ* are constants, and found *F*_max_ = 8.3 *α*S min^−1^, *τ*  = 1.9 min (*R*^2^ = 87%) (resp. *F*_max_ = 2.8 *α*S min^−1^, *τ*  = 0.4 min (*R*^2^ = 97%)) for the group with (resp. without) IES. To conclude, this result shows that *α*S events are three times more frequent in group with IES compared to the other group during induction and maintenance.

Finally, the third variable that we considered is the changes in *S*_*α*_ averaged amplitude, computed as the mean area *A*_*α*_ and *A*_*IαS*_ below the curve |*S*_*α*_(*t*)|, estimated for the *α*S and I*α*S events (Fig. 2d, e). During the induction phase, the area *A*_*α*_ decreased for patients with IES (magenta) with an initial slope *γ* = −8.1 nV min^−1^ while it was almost constant (*γ* = −0.5 nV min^−1^) for the other group (gray) (Fig. 2d). In contrast, the power of the signal *S*_*α*_ did not separate the two groups (Supplementary Fig. 2). The previous analysis applied to the inter-*α*S signal by computing the area *A*_*IαS*_ for both populations almost superimposed and thus could not be used to separate each population (Fig. 2e). We conclude that *α*S and I*α*S evolve quite differently, in particular, the decrease of the *α*S amplitude can be used to separate the two groups of patients, which was not the case for I*α*S.

To conclude, the three variables: duration Dur_*α*_, occurrence frequency *F*_*α*_ and average amplitude *A*_*α*_ evaluated during the induction phase could be used to determine the class to which a patient belong to. Additionally, for the IES group, these variables reached a steady-steady state with a time scale similar or shorter than IES proportions *P*_IES_ (Fig. 1g, red and Supplementary Fig. 2). These results confirm that these variables are good candidates for predicting IES.

### Predicting IES from the induction phase

During surgery several variables are routinely collected to monitor anesthesia. The Mean Arterial Pressure (MAP) is acquired continuously to prevent propofol hypotensive effect (among others), while age, weight and height are used in setting the amount of intravenous analgesia and anesthetic agents^21^ (chap. 8). Additionally, we also included in the analysis the per-operative MAP drop (ΔMAP) as its link with post-operative complications is still debated^22,23^. We explored here the capacity of these non-EEG variables and the ones extracted from the alpha-band *S*_*α*_ signal to predict the appearance of IES during the first 35 min of the surgery, based only on the first 10 min.

**Fig. 3:**
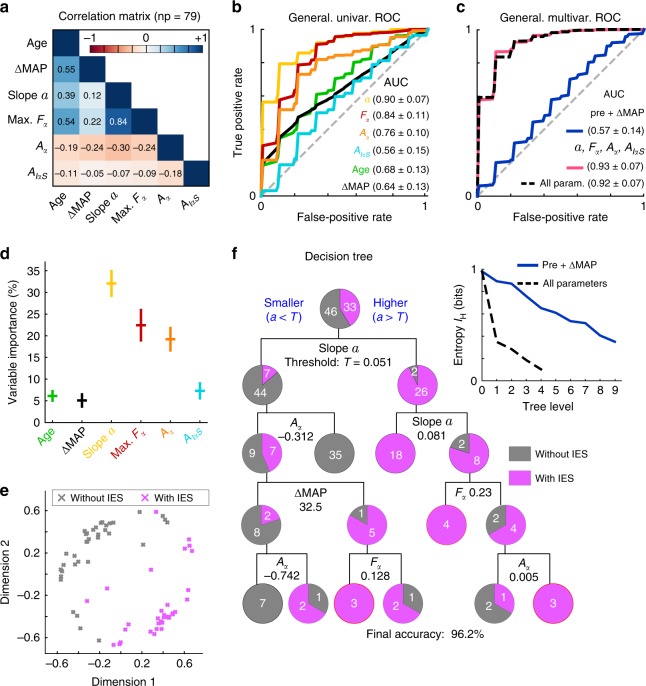
IES prediction capabilities of the proposed features. **a** Pearson’s correlation matrix for age and per-operative (ΔMAP, slope *a*, *F*_*α*_, *A*_*α*_, *A*_*IαS*_) features computed on *np* = 79 patients. **b** Generalization ROC curves of obtained from a nested-cross-validation scheme (see method) applied to univariate logistic regression classifiers based on the individual features presented in (**a**). **c** Generalization ROC curves computed as in (**b**) but for multivariate random forest classifiers based on the *αS*-related features (red) or the entire features set (dashed black). **d** Average (and STD) Feature importance computed from the individual RF classifiers trained on the entire features set from (**c**). **e** Two dimensional representations of the distance metric induced by the optimal RF classifier using only *S*_*α*_-related variables. **f** Optimal (see method) classification tree trained on the entire dataset presenting the ability of the *α*-related features to separate IES (pink) from non-IES (gray) patients. **f** inset: information entropy *I*_*H*_ (see method) as a function of the tree level (number of decisions taken) for the decision tree using all variables presented in (**e**) (dashed curve) and the tree based only on non-EEG variables presented in Supplementary Fig. 5

First, we looked at the correlation between each pair of variables. We computed the Pearson-correlation matrix (Fig. 3a), which showed the strongest correlation (*ρ* = 0.84) between the slope *a* (slope of *P*_*α*_) and the frequency *F*_*α*_, while the other variables (age, height, weight, ΔMAP, *a*, *F*_*α*_, *A*_*α*_ and *A*_*IαS*_) were not or weakly (−0.32 ≤ *ρ* ≤ 0.39) correlated with each other, except for the age that was moderately correlated with *F*_*α*_ (*ρ* = 0.54) and ΔMAP (*ρ* = 0.55). Mostly weak correlations of *S*_*α*_-related variables with each other as well as with the non-EEG variables, indicates that they represent different aspects of the signal and therefore justify their use in classification.

Then, we evaluated the ability of these variables to predict the appearance of IES during the first 35 min of the surgery using only the induction phase (first 10 min). We started by testing individual variables, using univariate Logistic Regression (LR) classifiers (Fig. 3b). To train and evaluate the performance of the different classifiers, we used a nested k-fold cross validation procedure combined to a grid-search optimization for finding optimal hyper-parameter values (see Supplementary Table 1). We found that the age and ΔMAP were poor classifiers, with average areas under the ROC curves area under the curve (AUCs) of 0.68 ± 0.13 and 0.64 ± 0.13 respectively. The *A*_*IαS*_ slope failed to predict IES with an average AUC of 0.56 ± 0.15, while the others were fair to good classifiers with AUCs of 0.76 ± 0.1, 0.84 ± 0.11 and 0.90 ± 0.07 for *A*_*α*_, *F*_*α*_ and *a* slopes respectively. Furthermore, the height and weight variables presented no separations between the classes (Supplementary Figs. 3 and 4) and were not used. At this stage, we conclude that the *S*_*α*_-related variables: slope *a*, *F*_*α*_, *A*_*α*_ (except for *A*_*IαS*_) are stronger predictors than the non-EEG variables: ΔMAP, age, weight and height.

We then evaluated the capacity of multivariate classifiers to improve classification accuracy. We considered three ensembles of Random Forest (RF) classifiers based either on: only *S*_*α*_-related variables (slope *a*, *F*_*α*_, *A*_*α*_, *A*_*IαS*_), only non-EEG variables (age, weight, height and ΔMAP) or all variables and used the same training/evaluation procedure as for the univariate classifiers (see “Method”). The generalization ROC curves presented in Fig. 3c show that the classifiers using *S*_*α*_-related only and all variables obtained excellent average generalization AUCs of 0.92 ± 0.07 and 0.93 ± 0.07 respectively. The classifier based only on non-EEG variables failed to distinguish the two classes with an average AUC of 0.57 ± 0.14. We conclude that both in uni- and multivariate classifiers, the *S*_*α*_-related variables efficiently predict IES while non-EEG variables carry little predictive information.

We further confirmed the importance of *S*_*α*_-related variables for predicting IES by extracting the variable importance^24^ from the RF classifiers based on all variables presented in Fig. 3c. We found that the classifiers mostly used three variables: the *a* slope (32.1 ± 2.8% importance), the frequency *F*_*α*_ (22.4 ± 3.5%) and the *A*_*α*_ slope (19.2 ± 2.6%) (Fig. 3d), while the other variables all had importances below 8%. Lastly, we assessed the capacity of the best RF classifiers using *S*_*α*_-related only and non-EEG only variables to separate patients with and without IES. To this end, we computed the proximity plots, a two-dimensional projection of the distance induced by a random forest, presented in Fig. 3e for *S*_*α*_-related only and Supplementary Fig. 5 for non-EEG only classifiers. Comparing the two proximity plots clearly showed that the *S*_*α*_-related variables only classifier almost perfectly separated the two classes while for the non-EEG variables only RF classifier the two classes are mixed.

Finally to further characterize the structure of our 79 patients dataset, we optimized the hyper-parameters of a classification tree^25^ (p. 308) using all the variables to obtain the best accuracy score on the entire dataset (see method). This tree, presented in Fig. 3f, had only four levels and a prediction accuracy of 96.2% with the initial split using the *a* slope (threshold *T* = 0.051) and already allowing to mostly separate the two populations with an accuracy of 88.6%. We remark that only one split out of eight is based on a variable which is not *S*_*α*_-related. In contrast, a tree based only on non-EEG variables (age, weight, height, and ΔMAP) failed to correctly classify the two groups, with an accuracy after four splits of 73.4% (Supplementary Fig. 5). To finely assess the quality of the tree, we used an information entropy metric (see “Method”) to compute the purity of the tree at each level as presented in Fig. 3f inset. We compared the tree using all variables (Fig. 3f) with the one using only non-EEG variables (Supplementary Fig. 5) and found that the former is able to attain a better entropy in much fewer levels (*I*_*H*_ = 0.10 at tree level 4 compared to *I*_*H*_ = 0.35 at level 9).

## Discussion

We presented here a general method to detect, during GA, transients and partial suppressions in the alpha rhythm (*α*S), that we showed could be used to predict occurrences of iso-electrical suppressions. *α*S events are present in the EEG signal of both patients with and without IES, however their statistics were clearly different between the two groups and that already in the first 10 min of GA. In particular, patients that developed IES had in average more frequent and longer *α*S periods (Fig. 2). In addition, the average amplitude of *α*S events rapidly decreases during the induction phase for patients from the IES group. This property was specific to *α*S and contrasted with periods of no-suppressions in the alpha rhythm defined as inter-*α*S (I*α*S) time periods. Finally, the *α*S statistics, estimated during the first 10 min of anesthesia, could be used to efficiently predict whether patients will develop IES with an accuracy of 96.2% and a ROC AUC of 0.93.

Monitoring EEG during GA has become a routine practice, yet it remains difficult to interpret and predict. GA can result in mild cognitive impairments but also lead to devastating cognitive complications such as confusion or delirium, which might results in of loss of autonomy^7,11,26–29^.

A poor cognitive outcome has been associated with IES in EEG. To our knowledge, no previous methods have been proposed to either anticipate IES or to consider that *α*S is a predictive and reliable marker^7,18^. The method we developed here allows to anticipate IES and therefore to identify patients with a potential cerebral fragility.

Patients sensitivity to GA has been found to be closely related to IES appearances^30,31^. Therefore, by showing that patient propensity to develop IES can be assessed from the first minutes of induction, we propose here that patient sensitivity to GA could also be evaluated on that same time scale. We suggest that IES prediction evaluated from the early stage of GA combined with an *α*S tracking procedure could be used by practitioners to finely tune the anesthetic dose^32^. Another possible application of our method might be to identify factors, such as comorbidities, psychotropic treatments (bezodiazepine, antidepressant medication), linked to postoperative cognitive complications^7,33^.

We focused here on drug-induced alpha-band suppressions, characterized by the dominance of a delta rhythm in the EEG^34^. During GA, transient events occurring in the alpha rhythm are mostly described as ‘spindle-like’ EEG patterns resulting from propofol GABA-mediated inhibition^35–38^. Interestingly, the phase coupling between alpha spindle-like epochs and delta rhythms^19,37^ has been found to measure the degree of patient consciousness during GA. Although a profound loss of consciousness is associated to emergence of IES, this correlation analysis described in refs. ^19,37^ did not result in predicting IES occurrences. However, we showed here that suppression periods in the alpha rhythm, *α*S, contain enough information about GA to predict the appearance of a large number of IES and therefore estimate patient sensitivity to GA.

The depth of anesthesia can be quantified by several markers such as the delta band power or the alpha-to-delta ratio^39^. The performance of these markers in predicting the occurrence of IES during the first 35 min, can be obtained by comparing the *α*S’P_*α*_ proportion slope *a*’ to the alpha-to-delta ratio or the delta power. For that goal, we estimated these parameters during the induction phase. We started by computing the power spectrum during the induction phase for the groups presenting or not IES. We estimated the alpha-to-delta ratio (Supplementary Fig. 6) and found that the power spectrum between the two groups (with and with no IES) was visibly different. Using a univariate logistic model, we show here that *α*S features (’P_*α*_ proportion slope *a*’) outperformed alpha-to-delta ratio by 20% (85% vs 65%, respectively (table of Supplementary Fig. 6). Similarly, the *δ*-power had an accuracy of 73.75% vs 75% and 85% for *α*S amplitude (A_*α*_) and’P_*α*_ proportion slope *a*’. Interestingly, multivariate analysis shows that the *δ*-power and the *α*S amplitude are independent (see Supplementary Fig. 7). Yet, using these two variables, we found an accuracy of 76.25% and an AUC of 0.825 which remains much below the performance obtained with the’P_*α*_ proportion slope *a*’ variable alone. Finally, when we compare the *δ*-power and’P_*α*_ proportion slope *a*’, we found that there are not independent: in that case the *δ*-power was no longer significant (*p* = 0.363), while the ’P_*α*_ proportion slope *a*’ is clearly the most predictive variable with a *p*-value < 0.0001. These results support that *α*S are the most suited to predict IES occurrences. We discuss here three complementary correlations:

First, patients without IES during the first 35 min, developed few to no IES during surgery, as shown in Table 1. Still, in this study doses of propofol during the first 35 min were in average higher (Target Controlled Infusion, TCI = 4.3 μg ml^−1^) than during the rest of the surgery (TCI = 3.6 μg ml^−1^). We recall here that a higher TCI is associated with more IES^10^.

Second, the maximal mean arterial pressure drop (ΔMAP) measured during induction is neither correlated with *α*S nor with IES, as shown in Fig. 3. This point confirms recent findings showing that MAP drop and POCD are poorly correlated^23^, contrary to IES and POCD^7^. Additionally, to assess whether the lowest MAP during the induction period could predict or not IES, we used an univariate logistic model, and we found no significant correlation results with *p* = 0.848, AUC = 0.51. This is in contrast with The ΔMAP that was significant with *p* = 0.026, AUC = 0.64 with a classification accuracy of 66.25% (vs. 58%).

Third, we found here that patient age poorly predicted IES occurrences, as shown in Fig. 3. This absence of prediction was more noticeable since patients more than 65 years old were expected to be more sensitive to GA^7,33^. Nevertheless, in the present study the 10 youngest patients (<29.5 yrs) did not develop IES during GA. Yet, patients from the IES group (age mean ± SD: 59.2 ± 14.3 yrs) were thus not all necessary (>65 yrs), suggesting therefore that the age criterion should be reconsidered for the evaluation of patient GA sensitivity. Finally, to clarify that during the maintenance, IES increase cannot be attributed to an increase of propofol, we compare the averaged TCI for groups of patients with no IES vs IES (Supplementary Fig. 8): we find that the no IES group had a slightly higher TCI during maintenance compared to the IES group (median [IQR)]; TCI = 3.7[3.3, 4] μg ml^−1^ ‘no IES’ vs 3.2[3, 3.6] μg ml^−1^ for ‘IES’ patients). This result could be understood by an adaption of the propofol dose to the patients response. This result confirms that, propofol TCI cannot be used to predict the appearance of IES.

The predictive power of *α*S statistics described here for the IES was tested for anesthesia induced with propofol. Different anesthetics such as halogenated gases also trigger loss of consciousness, but they can involve different neuronal mechanisms^40^. These inhaled anesthetics can induce BS activity and thus IES through a GABA-mediated inhibition^41^. Consequently, for GA based on volatile anesthetics, the same causality between alpha-rhythm activity and IES could exist. In particular, we suggest that *α*S generically anticipates IES.

Similarly, it is unclear whether the present method could be used with GA based on NMDA antagonizing drugs, such as ketamine, nitrous oxide, and xenon that tend to enhance arousal, resulting in the increase of the overall EEG activity^42,43^ or on the contrary, leads to paradoxical IES occurrences increase, when used in combination with classical anesthetics^44^. Furthermore, we focused here on alpha and delta rhythms, which are the most relevant for propofol-induced GA^15^. Yet, other frequencies such as beta and gamma bands, also present during GA, could be used to clarify the neurophysiological mechanisms underlying loss of consciousness, in particular the role of thalamus^45–47^.

Our results show that detecting the arrival of transient *α*S events allows us to predict patient risk of developing IES. However, it remains to be demonstrated if a drug administration guided by our *α*S analysis would reduce the occurrence of IES and effectively prevent post-operative cognitive dysfunctions^33^. Further studies are therefore needed to confirm these hypotheses. Although patients with poor post-operative outcomes present more IES during their anesthesia^7^, no causal relations have been found between IES and complications following GA. Post-operative complications and IES could both arise from pre-existing pathologies or cerebral fragility. Further investigations are clearly needed to clarify how per-operative events (IES and *α*S) translates into post-operative consequences (worsening of mild cognitive impairments, post-operative delirium, etc)^8,18^. Finally, we analyzed here an EEG signal free of artifacts resulting from head manipulation during surgery. Nevertheless, it would be interesting to automatize the artifact detection/correction stage, similarly to^48^.

In the present study, we found EEG features to predict periods of IES from the first minutes of a GA. We obtained statistics of partial suppressions of the alpha rhythm, and showed that they were better predictor of IES than classical variables such as the age and the ΔMAP. We showed that *α*S features can be used successfully to classify patients that will develop IES during GA. Partial *α*S, and in particular their causal link to IES, could hopefully lead to a better understanding of GA.

## Methods

### Ethics statement

This prospective observational study included patients receiving a scheduled elective procedure requiring GA at Lariboisière University Hospital (Paris, France). This study was approved by the Institutional Review Board of La Société de Réanimation de Langue Française (CE SRLF 11-356). Patients were excluded from the study if their age was <18 years or they underwent an emergency procedure. In agreement with the ethics committee for this non-interventional study an information letter was given, and an oral agreement was obtained from each patient before anesthesia.

### Participants

The group of 80 sedated patients (49 women, 31 men; mean age ± SD, 52.8 ± 17.5 yr) used in this prospective, observational, single-center, routine care study were all from the Lariboisière Hospital (Paris, France). Data were collected between January 2018 and May 2018. Patients where admitted for a non-emergency elective neuroradiology (*n* = 60) or orthopedic (*n* = 20) surgery (Table 2). Pregnant women, minor patients (<18 yr), patients with a body mass index (BMI) > 35 kg m^−2^ (invalid Schnider model^21^, (p. 23)) and patients sedated under mechanical ventilation at the time of their management were excluded from the study. The inclusion criteria for patients was to be anesthetized with a Total IntraVenous Administration (TIVA) of propofol Target Controlled Infusion (TCI) according to the Schnider model, and combined with a morphine derivative (remifentanil or sufentanil)^21^, (chap. 8).

**Table 2:** Study group per-operative and demographic data

Nb. of patients	*np* = 80
Age (mean ± SD, range)	52.8 ± 14.5, 18–85 yr
Gender ratio, female/male	1.42
Surgery (Ortho./Neuro.)	20/60
Height (mean ± SD)	167 ± 8 cm
Weight (mean ± SD	70.2 ± 13.6 kg
BMI (mean ± SD)	25.2 ± 4.5
Induction Dur. (mean ± SD)	9.5 ± 2.7 min
Max. MAP drop (Ind.)	28.3 ± 10.6 mm Hg

### GA induction, maintenance, and monitoring

GA was induced in a standardized manner with a morphine derivative followed by intravenous administration of propofol. Patients from neuroradiology surgery group received remifentanil as a morphinic agent with, a TCI ranging from 5 to 6 ng ml^−1^ (Minto model^21^ (chap. 2, 10)) until oro-tracheal intubation, the dose then decreased to 3 to 3.5 ng ml^−1^ during maintenance, depending on the anesthesiologist’s decisions in the operating room.

For patients included in orthopedic surgery, the morphine used was sufentanil in iterative administration (bolus between 5 and 15 gamma), under the responsibility of the anesthesiologist in charge of the patient.

The brain TCI for propofol, during induction, was 5 μg ml^−1^ according to the Schnider model (until oro-tracheal intubation)^21^ (chap. 2, 10). If patients showed signs of arousal after several minutes at the target concentration, the anesthesiologist could increase propofol concentrations.

All patients were intubated after curarization by atracurium besilate (0.5 mg kg^−1^, Atracrium®) and mechanically ventilated with a tidal volume of 6–8 ml kg^−1^ and a respiratory rate adapted to obtain an EtCO2 between 35 and 38 mm Hg. After intubation, the anesthesiologist was instructed to maintain the patients’ PSI between 25 and 40. The administration of fluids and vasoconstrictors was left to the discretion of the anesthesiologist present in the operating room based on standard care protocol of our institution.

Standard monitoring (Pulse Oxygen Saturation: SpO2, Heart Rate: CF, Systolic and Diastolic Blood Pressures (SBP, DBP) as well as Mean Arterial Pressure MAP, temperature, expired CO_2_ fraction: EtCO_2_) was associated with frontal EEG monitoring by the Masimo Sedline®monitor (electrodes: F7-Fp1-Fp2-F8, original sampling frequency: 2500 Hz).

### Statistics and reproducibility

We tested that the first *α*S and IES (Fig. 1f) times followed a Log-normal distribution using Kolmogorov-Smirnov (K-S) test, where we obtained for *α*S (K-S stat. = 0.099, two-tailed *P*-value = 0.778) and (0.097, 0.797) for IES. We then applied logarithm transform to obtain a Normal distribution that was tested with a Shapiro-Wilk (S-W) test where we obtained for *α*S (S-W stat. = 0.970, two-tailed *P*-value = 0.346 > 0.05) and (0.984, 0.812) for IES.

We identified and discarded outliers using Grubbs test with a 5% significance level and 99% of confidence interval on the *P*-value. We used a paired *t*-test to generate a parametric two-tailed *P*-value where the alternative hypothesis was ln(*t*_*α*−*S*_) < ln(*t*_*α*−*S*_) + ln(5) (*P*-value < 0.0001).

We tested difference in slope coefficient *a* distributions (Fig. 1h) for patients with and without IES using Mann–Whitney test that was used to generate a non-parametric P-value for a significance level 0.05, where we found for alternative hypotheses *a*_*αS*_ < *a*_IES_, *a*_*αS*_ ≠ *a*_IES_, and *a*_*αS*_ > *a*_IES_ the *P*-values (one-tailed) <0.0001, <0.0001, and 0.9999 respectively.

### Detection of IES and *α*S

**Fig. 4:**
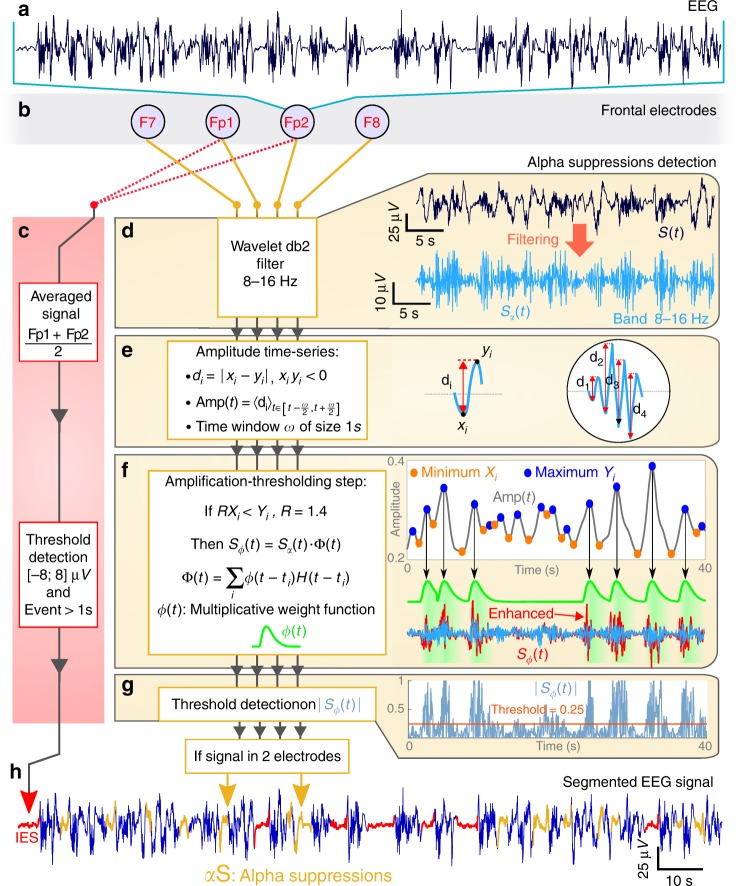
Alpha-Suppression (*α*S) detection procedure. **a** Unsegmented EEG signal. **b** Frontal electrodes. **c** Detection of IES based on Fp1 and Fp2. Box 1: signal average (Fp1 + Fp2)/2. Box2: threshold detection for regions inside ±8 μ*V* band, lasting at least 1 s. **d** (Upper) EEG signal *S*(*t*) wavelet decomposition filtered in the band 8–16 Hz resulting in *S*_*α*_ (light blue). **d** (Middle) Extraction of oscillatory features of *S*_*α*_(*t*): the minimums *x*_*i*_, the maximums *y*_*i*_, the distance *d*_*i*_ = |*x*_*i*_ − *y*_*i*_|. The average Amp(*t*) of the distance *d*_*i*_ is computed in the widow interval [*t* − *w*/2;*t* + *w*/2], where *w* = 1*s*. **d** (Middle low) Nonlinear filtering enhancing transient oscillatory periods. *X*_*i*_ (orange) and *Y*_*i*_ (blue) are Amp(*t*) (gray) minimums and maximums respectively. The multiplicative function Φ(*t*) is the sum of weight function *ϕ*(*t*) (green), multiplied by a Heaviside function *H* at time *t*_*i*_ of maximal amplitude *Y*_*i*_ = Amp(*t*_*i*_). The resulting function with enhanced oscillatory regions is *S*_*ϕ*_(*t*) = *S*_*α*_(*t*)Φ(*t*) (red). **d** (Lower) Signal below a *T* = 0.25 threshold (orange) applied on normalized |*S*_*ϕ*_(*t*)| (blue) and present at least on two electrodes is considered as an *α*-S. **e** Segmented EEG signal with detected IES (red) using method (**c**) and *α*-S (yellow) using (**d**)

The *α*S monitoring starts following propofol injection, with a loss of beta activity and an increase in the *α*-band. We then used the EEG signal collected from electrodes: Fp1-Fp2 to detect regions of IES, characterized by a small signal amplitude in the range [−8;8] μV that lasted at least 1 s (Fig. 4a–c)^49,50^.

To detect regions of *α*-Suppressions time periods, we designed a novel algorithmic procedure that consists in five steps.

For the first step, we used the Daubechies wavelet decomposition with two vanishing moments (db2) to filter the EEG signal *S*(*t*) in the band [8, 16] Hz that we assimilated to the alpha band^51,52^ (chap. 6). We have used the second level detail coefficient d2 (Supplementary Fig. 9). The resulting filtered signal *S*_*α*_(*t*) is shown in Fig. 4d (light blue). In the second step, we estimated from the signal *S*_*α*_(*t*) the minimums *x*_*i*_ and the maximums *y*_*i*_ conditioned that *x*_*i*_*y*_*i*_ < 0 (Fig. 4d).

1 $${\mathrm{Amp}}(t) = \frac{1}{w}\mathop {\sum}\limits_{i = 1}^{n_d(t)} 11_{S_\alpha \left( {[t - \frac{w}{2},t + \frac{w}{2}]} \right)}(x_i,y_i),$$

where *n*_*d*_(*t*) is the number of distances *d*_*i*_ falling inside the window $[t - \frac{w}{2},t + \frac{w}{2}]$ (Fig. 4d, gray).

In the third step, we designed a nonlinear filter to enhance the amplitude of transient oscillatory EEG regions present in the filtered signal *S*_*α*_(*t*). Using the minimums *X*_*i*_ and maximums *Y*_*i*_ of the function Amp(*t*) (Eq. (1)), we identified the ensemble (*X*_*i*_, *Y*_*i*_) satisfying *RX*_*i*_ < *Y*_*i*_ (*R* = 1.4). For each selected *i*, we recover the times sequence {*t*_*i*_} such that Amp(*t*_*i*_) = *Y*_*i*_. We defined the amplification function Φ as the sum of the function

2 $$\phi (t) = 1 + B\left( {\frac{t}{{t_b}}} \right)^\eta e^{\eta (t/t_b - 1)},$$

(*B* = 0.72, *t*_*b*_ = 1*s* and *η* = 1.96 are constants) positioned at *t*_*i*_ and truncated by an Heaviside function *H*, so that

3 $$\Phi (t) = \mathop {\sum}\limits_i \phi (t - t_i)H(t - t_i).$$

Note that *ϕ* is maximal at *t*_*i*_. Finally, the enhanced signal is given by

4 $$S_\phi (t) = S_\alpha (t)\Phi (t),$$

which represents the filtered signal *S*_*α*_(*t*) where non *α*S-regions have an amplified amplitude (Fig. 4d, red).

In the fourth step, to find the *α*S regions we applied a threshold *T* = 0.25 to the normalized *S*_*ϕ*_(*t*) (by the maximum over the sliding window) and searched for regions where $|\bar S_\phi (t)| < T$ (Fig. 4d). To repairs noise-induced breaks in *α*S events we apply a dilation of 0.9 s, then an erosion of 1.1 s to discard too short *α*S.

In the fifth step, we detected an *α*S periods for each electrode. We retain an *α*S, if it is at least present on two of the four frontal electrodes. Note that *α*S represents partial signal suppressions occurring in the [8, 16] Hz band. The result of the IES and *α*S detections is shown in Fig. 4e.

### Moving window analysis

From the segmented EEG signal (Fig. 4e), we used a 24*s* time window *W*_*t*_ to identify the proportion *P*_*α*_ (resp. *P*_IES_) of signal spent in *α*S (yellow) (resp. IES (dashed red)) state (Avg. in Fig. 1d, e, g and individual patient traces in Supplementary Fig. 1). Note that we analyze the signal before time *t* = *t*_eval_ (Fig. 1e, green dot). After each step the window is moved by 1 s.

### Time of appearance of the first *α*S and IES events

We estimated the first time *t*_*αS*_ (resp. *t*_IES_) that *α*S (resp. IES) appears in the EEG signal. For that goal we look for the first time that a yellow (resp. red) region appears. To guaranty the robustness for the detection of the times *t*_*αS*_ (resp. *t*_IES_), we used the criteria that the total duration of the *α*S events (resp. IES) exceed at least 5% of the 240 s time duration window.

### Properties of *α*S at the initial time

We constructed the curve *P*_*α*_ that represents the proportion of time *α*S spent in the moving window *W*_*t*_ (Fig. 1g, yellow). We introduce the slope *a* at time zero of *P*_*α*_ as a statistical parameter. To estimate *a*, we fitted *P*_*α*_ with equation *y* = *at* for the first 10 min (Fig. 1g, dashed blue).

### Estimation of *α*S event duration

From individual segmented EEG signal (Fig. 4e), we constructed a time-series Dur_*αS*_(*t*) of *α*S durations where for each detected time point we collected the duration. To construct an average (Fig. 2b) over patient population, we use a linear interpolation between the detected time.

### *α*S occurrence frequency

We evaluated the number of *α*S events per minute that we denote *F*_*α*_(*t*) by counting within a moving time window of size *w*_*f*_ = 50*s* the number (Nb.*α*S) of distinct *α*S in the segmented EEG signal:

5 $$F_\alpha (t) = \frac{{|\# \alpha {\mathrm{S-events}}[t - w_f/2{\mkern 1mu} ,{\mkern 1mu} t + w_f/2]|}}{{w_f}}.$$

The time window moved 10 s forward after each estimation.

### Average amplitude per *α*S events

From individual filtered signal *S*_*α*_(*t*), we constructed a time-series *A*_*α*_(*t*) of the average amplitude defined as the area under the curve |*S*_*α*_(*t*)| averaged over each *α*S events duration $T_\alpha ^jS$. For each *j*^th^ *α*S events starting at $t_i^j$ and ending at $t_f^j$:

6 $$A_\alpha (t_j) = \frac{1}{{|T_{\alpha S}^j|}}{\int}_{T_{\alpha S}^j} {|S_\alpha (s)|} {\mkern 1mu} \mathrm{d}s,$$

where $T_{\alpha S}^j = [t_i^j,{\kern 1pt} t_f^j]$. The same method was applied to estimate time-series *A*_*IαS*_(*t*) obtained from the *S*_*α*_(*t*) area under each inter-*α*S regions (Fig. 2a, blue). Fitting was performed using’fit’ function in MATLAB R2018a.

### Reporting summary

Further information on research design is available in the Nature Research Reporting Summary linked to this article.

## Supplementary information

Supplementary Information

Description of Additional Supplementary Files

Reporting Summary

Supplementary Data 1

## Data Availability

Data are available from the authors upon reasonable request.

## Code availability

The code is associated to a filled patent. The method is property of SignalMed+. 
