# Supplementary material for: Alpha rhythm collapse predicts iso-electric suppressions during anesthesia
Source: Commun Biol. 2019 Sep 2;2:327. doi: 10.1038/s42003-019-0575-3 (PMC6718680; doi:10.1038/s42003-019-0575-3)
Supplement: Supplementary file 1 — Supplementary Information [file 42003_2019_575_MOESM1_ESM.pdf]

1 Supplementary Figures

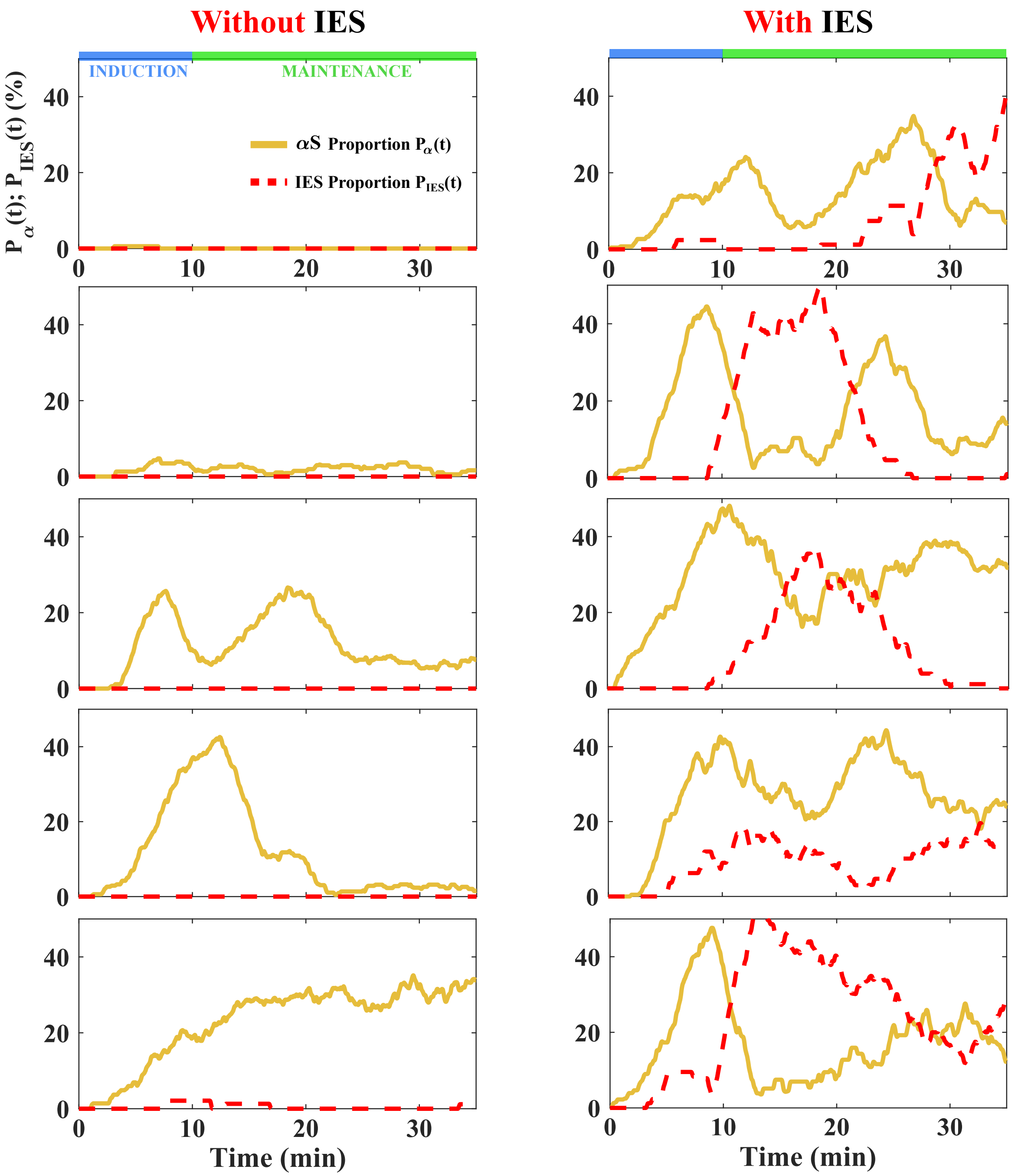

Figure 1: **Proportions P_α and P_{IES} for individual patients.** αS (resp. IES) proportion $P_\alpha(t)$ (yellow) (resp. $P_{IES}(t)$ (dashed red)) estimated for single patients without IES (left) and with IES (right) (See also Main text, Fig. 1D, E, G).

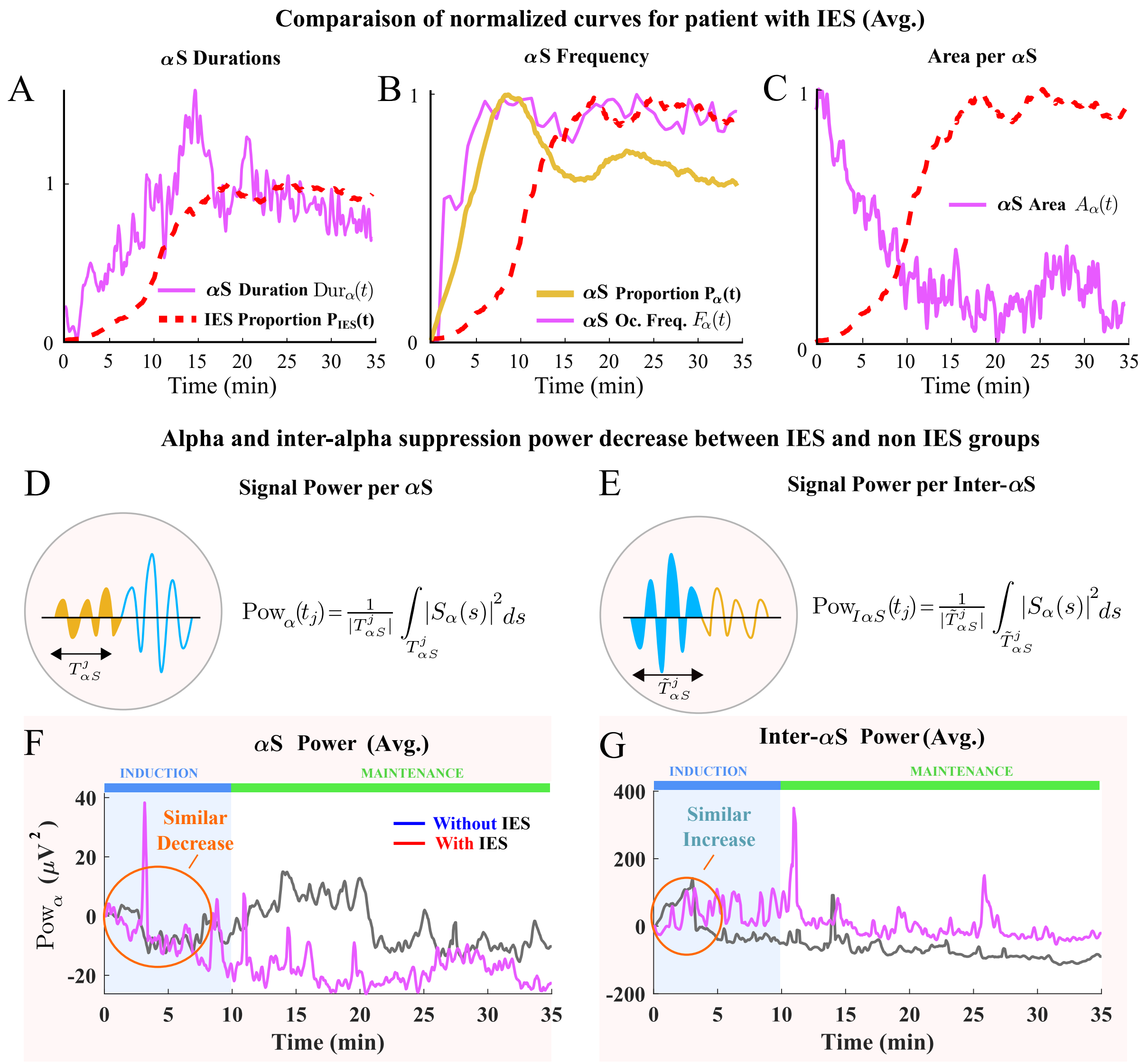

Figure 2: **αS variable and IES proportion time course A.** Comparison of αS duration Dur_α (magenta) (Fig. 2B) and IES proportions P_{IES} (dashed red)(Fig. 1G). **B.** αS occurrence frequency F_α (magenta) (Fig. 2C), αS proportion (yellow) (Fig. 1G) and IES proportions P_{IES}. **C.** Area per αS duration A_α (magenta) (Fig. 2D) and IES proportions P_{IES}. Curves from panels **A-C** has been normalized to their respective value averaged over $[20, 35]$min. **D-E.** Schematic representation of signal $S_\alpha(t)$ power estimated for an αS (yellow, **D**) and an Inter-αS (blue, **E**). **F-G.** Comparison between patient groups with (magenta) and without IES (grey) of the $S_\alpha(t)$ power time course of estimated for αS (**F**) and an Inter-αS (**G**).

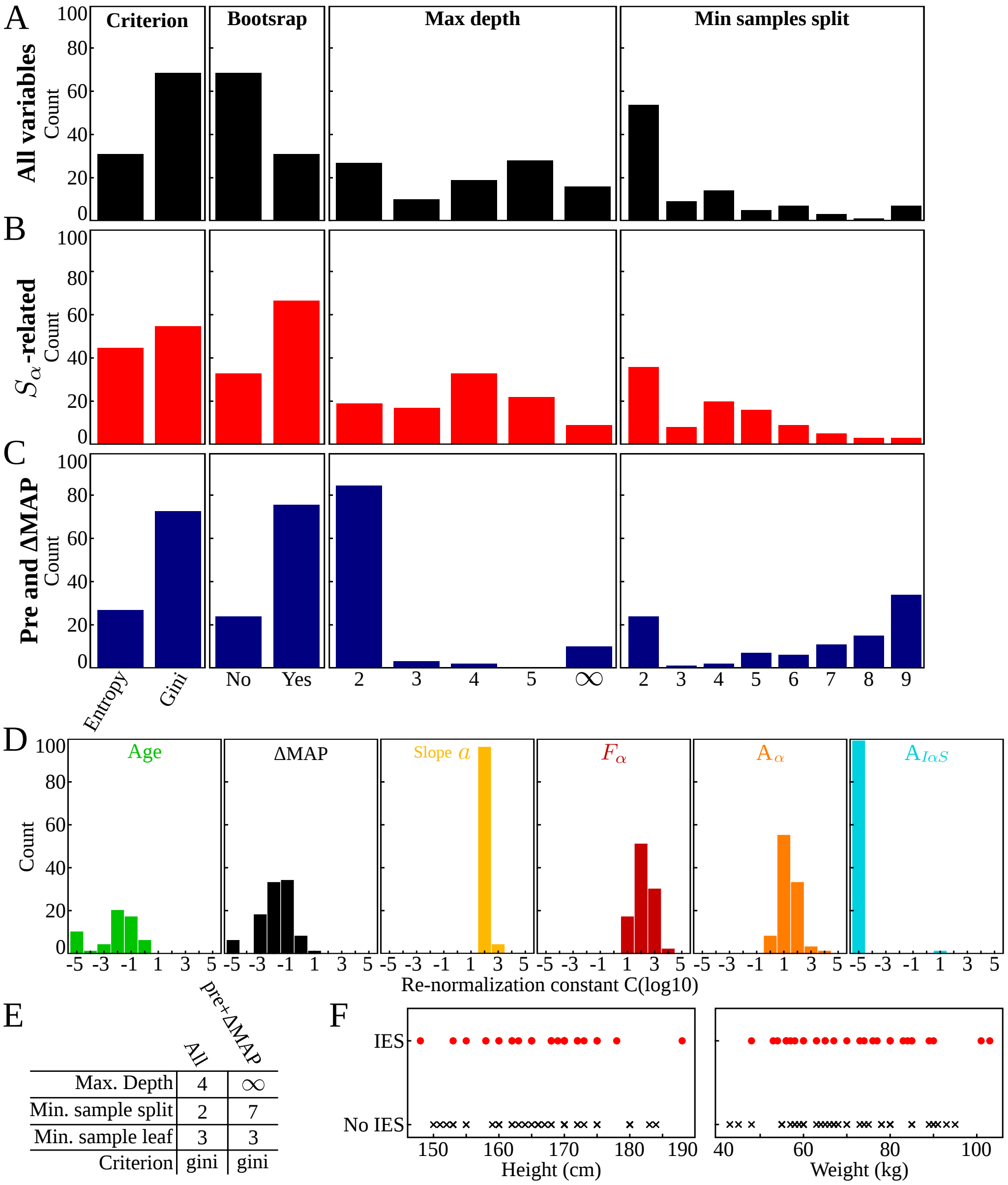

Figure 3: **Hyper-parameters and variable separability.** Random Forest (RF) classifiers hyper-parameters (Split criterion, bootstrap, maximal tree depth, minimum samples required for splitting a tree node) distributions for the 100 classifiers obtained by the grid search optimization under the repeated nested k-fold procedure for **A** the classifiers using all variables, **B** the classifiers using only S_α-related variables and **C** the classifiers using only age, height, weight and ΔMAP variables. **D** Hyper-Parameter (re-normalization constant C) distributions for the 100 uni-variate logistic regression classifiers obtained by grid search optimization under the repeated nested k-fold procedure for each considered variable: Age, ΔMAP, slope a, F_α, A_α and $A_{I\alpha S}$. **E** Optimal hyper-parameters obtained for the classification trees using either only pre-clinical and ΔMAP or all variables. **F** Distribution of the height and weight variables for the 79 patients depending on their class (IES and non-IES) showing non-separability of the two classes for these variables.

A

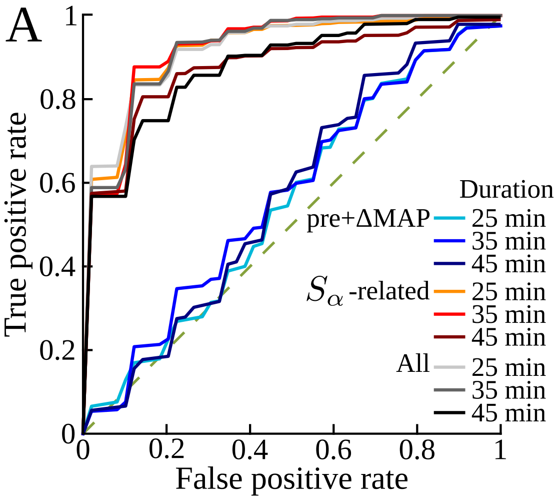

B

Classifier variables	Duration (min)	AUC AVG±STD
pre+ΔMAP	25	0.55±0.13
	35	0.57±0.15
	45	0.57±0.12
S_α-related	25	0.92±0.07
	35	0.93±0.06
	45	0.88±0.08
All	25	0.93±0.07
	35	0.92±0.06
	45	0.88±0.07

Figure 4: **ROC curves associated to RF classifiers where IES are evaluated in various time windows A** ROC curves for different RF classifiers obtained varying the time window in which IES are detected: 25, 35 (as presented in the main text) and 45 minutes. **B** AUC values associated to the ROC curves presented in **A**.

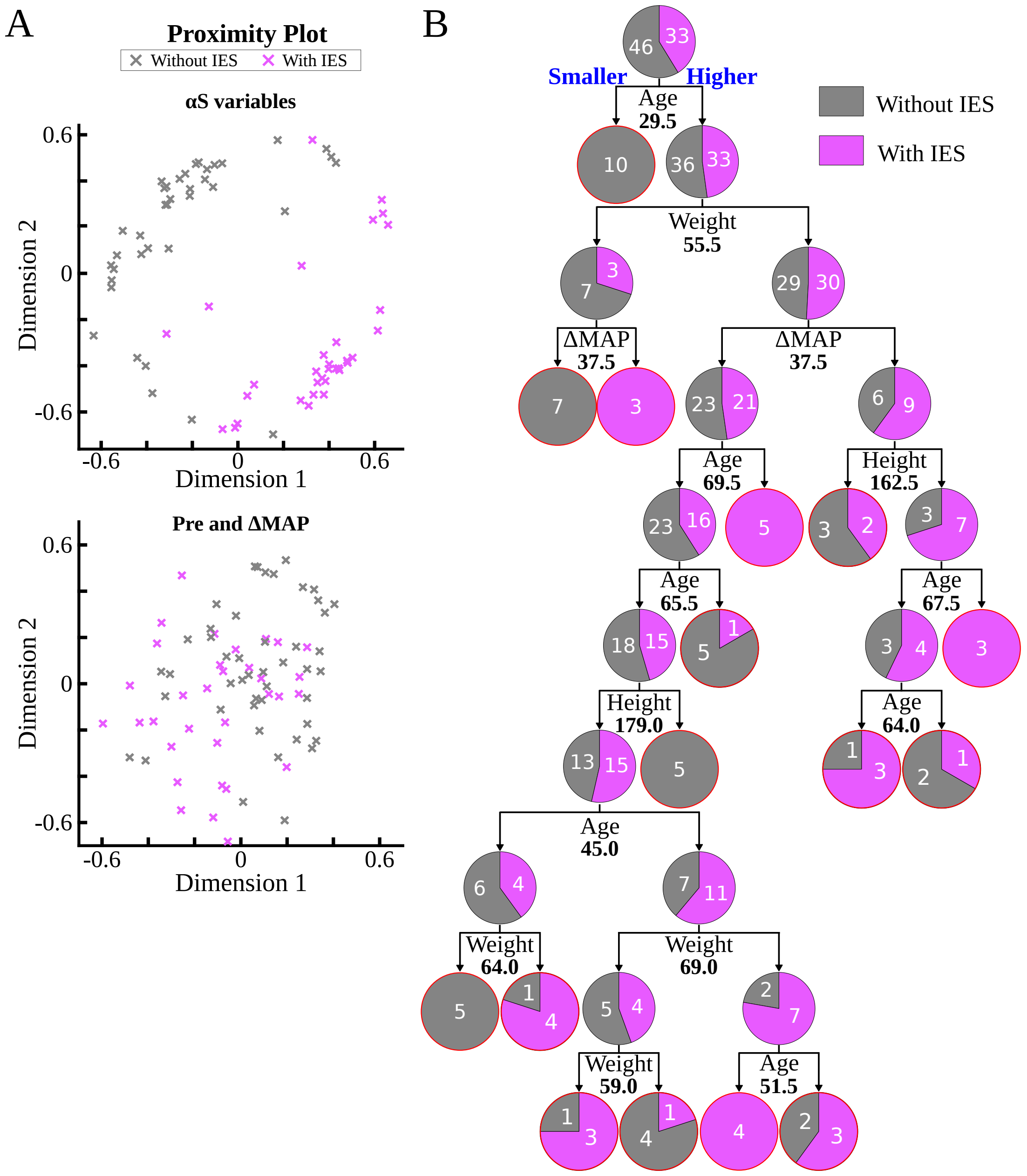

Figure 5: **Proximity plots and classification tree based on non-EEG variables A.** Two dimensional representations of the distance metric induced by the optimal RF classifiers using either only S_α-related variables (top, same as Fig. 3E) or only pre-clinical and ΔMAP variables (bottom). **B.** Optimal classification tree obtained from a similar procedure as the tree presented in Fig. 3F but using only pre-clinical and ΔMAP variables.

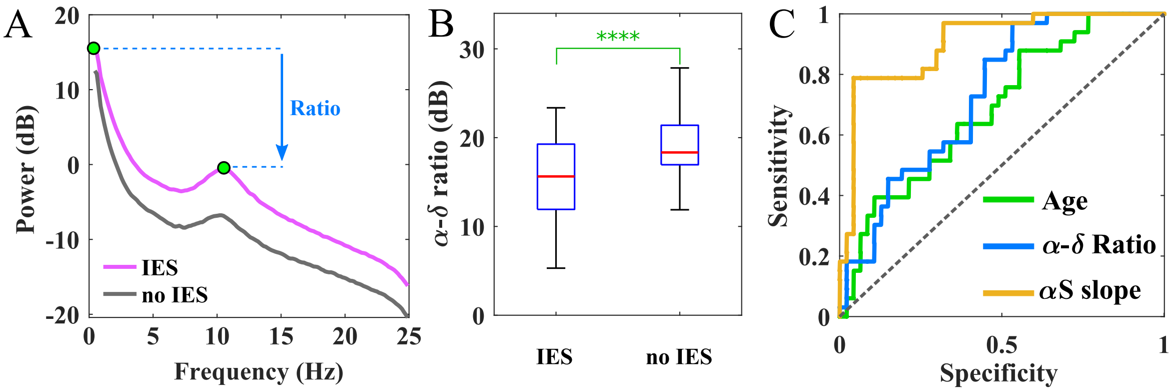

D **Univariate classification (Logistic)**

Variables	p-value	OR [95% CI]	AUC	Classification Accuracy
Age	0.008	1.041 [1.011; 1.071]	0.676	61.25%
α-δ Ratio	0.021	1.127 [1.019; 1.247]	0.721	65%
αS slope	<0.001	1.775 [1.412; 2.232]	0.901	85%

E **Correlation matrix (Pearson):**

Variables	Age	α-δ Ratio	αS slope
Age	1	**0.421**	**0.394**
α-δ Ratio	p<0.001	1	**0.368**
αS slope	p<0.001	p<0.001	1

Figure 6: **IES prediction (first 35min) based on Alpha to Delta Ratio (ADR).** **A.** Power spectral density computed over the induction period and averaged for the IES (magenta) and no IES (grey) groups. **B.** Distribution of ADR between the IES and no IES groups. **C.** Univariate ROC curves (logistic model) for predicting IES occurrence during the 35 first min using the age (green), ADR (blue) and the αS slope (yellow). **D.** Table summarizing model parameters from panel C. **E.** Correlation matrix between the age (green), ADR (blue) and the αS slope (yellow).

A

Univariate Logistic model

Variable	p-value	OR [CI 95%]	AUC	Classification Accuracy
**δ-power**	0.004	0.816 [0.709; 0.938]	0.734	73.75%
**αS amplitude**	0.001	0.269 [0.123; 0.586]	0.767	75%
**$P_\alpha$proportion slope 'a'**	< 0.0001	1.775 [1.412; 2.232]	0.901	85%

B

Correlation matrix (Pearson)

Variables	Age	$P_\alpha$proportion slope 'a'	αS amplitude	δ-power
**Age**	**1**	**0.394**	-0.192	**-0.369**
**P_α proportion slope 'a'**	p < 0.001	**1**	**-0.298**	**-0.364**
**αS amplitude**	p = 0.088	p = 0.007	**1**	0.091
**δ-power**	p = 0.001	p = 0.001	p = 0.420	**1**

C

Multivariate analysis (logistic)

Variable	p-value	OR	OR CI 95%
**αS amplitude**	0.002	0.271	[0.119; 0.17]
**δ-power**	0.014	0.831	[0.718; 0.963]

Figure 7: **Statistics of IES prediction (first 35min) based on the δ-power A.** Univariate analysis (logistic model) for the δ power (blue), the αS amplitude (red) and the P_α proportion slope a (yellow). All predictors are evaluated during the induction period. **B.** Correlation matrix for variable shown in table A. **C.** Significance analysis obtained from a multivariate logistic regression with two predictors: αS amplitude (red) and the δ power (blue).

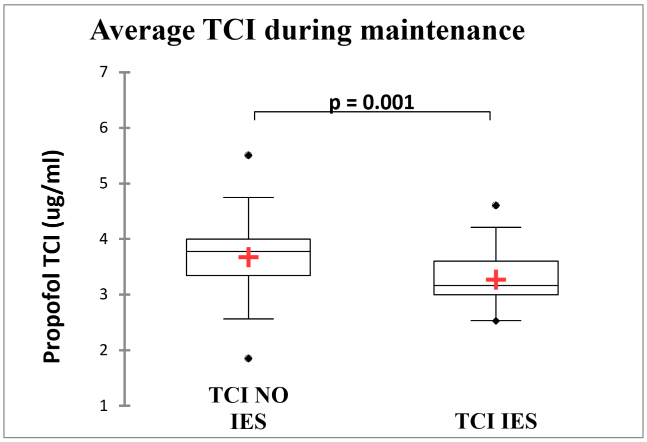

Figure 8: **Distribution of the averaged Propofol TCI for patient with and without IES.** The distribution is estimated during the entire maintenance phase of GA.

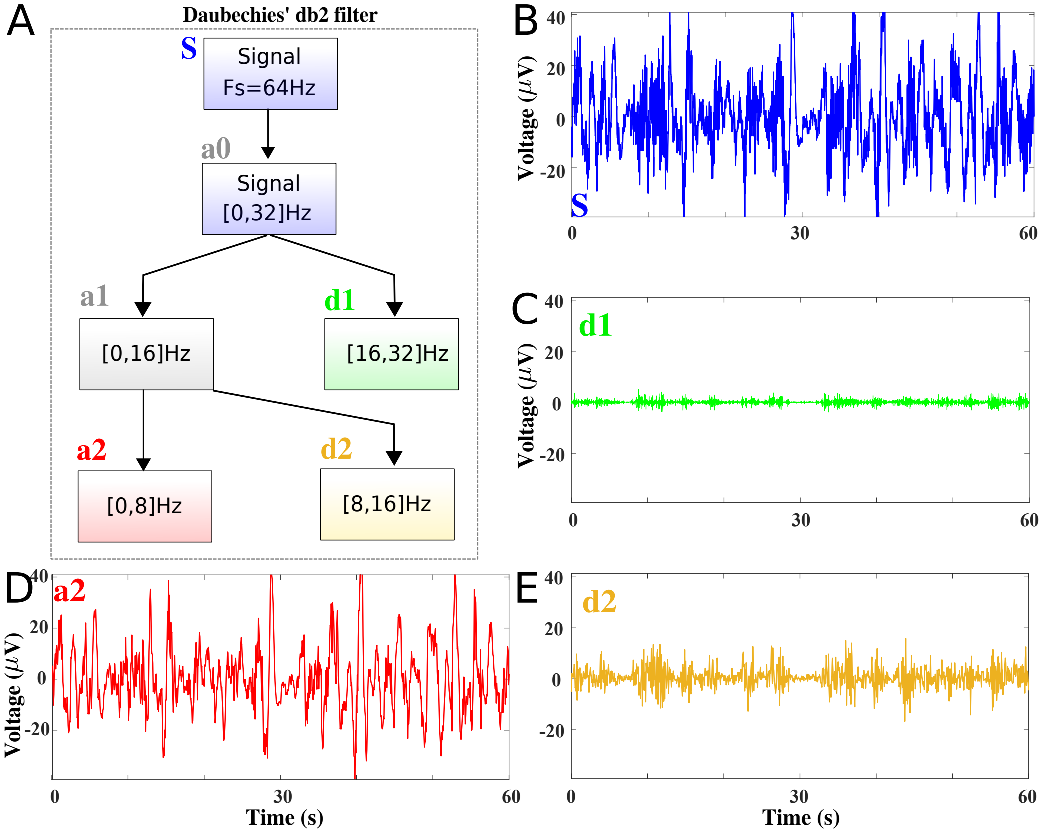

Figure 9: **Daubechies wavelets decomposition of the EEG Signal A.** Daubechies wavelet subband filtering scheme for an input signal S initially sampled at 64Hz, where $a0$, $a1$, $a2$ are the first approximation coefficients and $d1$, $d2$ the two first detail coefficients [2]. **B.** Unfiltered EEG signal. **C-E** Filtered signals for subbands $[16, 32]$Hz (**C**), $[0, 8]$Hz (**D**) and $[8, 16]$Hz (**D**).

2 Supplementary Tables

**Model**	**Hyper-parameter**	**Range**
**Logistic Regression**	Renormalization coef. C	10^k, $k = [\![-5, 5]\!]$
**Random Forest**	Max. depth	$2, 3, 4, 5, \infty$
	Min. sample split	$[\![2,\ 9]\!]$
	Bootstrap	Yes / No
	Criterion	Gini / Entropy
**Classification Tree**	Max. depth	$2, 3, 4, 5, 6, 7, \infty$
	Min. sample split	$[\![2,\ 11]\!]$
	Min. sample leaf	$[\![3,\ 7]\!]$
	Criterion	Gini / Entropy

Table 1: Hyper-Parameter ranges used in the grid-search optimization

3 Supplementary Notes, Discussion, Methods

3.1 Classification analysis

3.1.1 Classification variables

To classify patients with and without IES, we constructed classification variables computed from monitored MAP and alpha-band statistics. We defined the MAP variable as the difference between the reference MAP_{ref} (measured before GA) and the minimal MAP (estimated during induction): $\Delta\text{MAP} = \left|\text{MAP}_{ref} - \min_{10min}(\text{MAP})\right|$. The four classification variables related to αS events statistics were 1)the slope γ of P_α (named a hereafter), 2)A_α and 3)$A_{I\alpha S}$ estimated with $y(t) = \gamma t + b$ (b is a constant) on the first 10min, while for 4) the occurrence frequency F_α, we took the maximum evaluated over the same period.

3.1.2 Random Forest and Logistic Regression training procedures

For the training and evaluation of the generalization capabilities of the Logistic Regression (LR) and Random Forest (RF) classifiers, we used a stratified k-fold (k=5) cross-validation scheme (stratification ensures a balance between classes in each folds) on the $np = 79$ patients dataset.

To set the classifiers parameters, the hyper-parameters, we used a grid search optimization under a nested stratified k-fold cross-validation scheme [1], where $k = 5$ for both inner (training/validation) and outer (generalization) cross-validations. The hyper-parameter ranges used for each type of classifier are presented in Supplementary Tab. 1 and the resulting parameter distributions in Supplementary Fig. 3A-E. To reduce the variance resulting from the random splits occurring in the cross-validation procedure, we repeated the outer cross-validation 20 times and the inner cross-validations 10 times, which resulted for each setup in an ensemble of 100 classifiers. The reported ROC curves and AUC values (Fig. 3B-C, Supplementary Fig. 4) correspond to an average over the 100 individual ROC curves.

3.1.3 Random Forest validation

To assess the goodness of the variables and RF models applied on our dataset, we computed two metrics: variable importance and two-dimensional proximity plot. The former, presented in Fig. 3D consists in counting the number of times a split in a tree of the forest uses a given variable weighted by the corresponding impurity decrease [4]. The later, presented in Fig. 3E, uses a MultiDimensionnal Scaling (MDS) algorithm to obtain a set of $2D$ coordinates for each patient such that their Euclidean distance respects the dissimilarity measure computed from the RF classifier [3, p.570]. In our nested k-Fold cross-validation setup, we reported the average and standard deviation of the individual variable importance values from the 100 trained RF classifiers, while we present the proximity plots obtained for each setup from the RF classifier with the highest AUC value.

3.1.4 Classification tree training procedure

The classification trees obtained in Fig. 3F and in Supplementary Fig. 5, were obtained using the Classification And Regression Trees (CART) algorithm applied to the entire dataset of patients. We used the same nested k-fold cross-validation procedure as for the other classifiers to find the optimal set of hyper-parameters with respect to the accuracy score, using a grid-search optimization (Supplementary, Tab. 1).

3.1.5 Classification tree quality metrics

To ability of the classification trees to correctly separate the two data classes (”pure” nodes, containing only one class), we used the information entropy measure:

$$I_h(T) = \sum_{c\in\{0,1\}} \sum_{l\in T} \frac{N_c(l)}{N_c} f_c(l) \log_2(f_c(l)) \tag{1}$$

where $c \in \{0, 1\}$ (0: no IES, 1: IES) represents one class, $l \in T$ is a terminal node of the tree T, $N_c(l)$ is the number of observations of class c at node l, N_c is the total number of observations of class c in the tree and $f_c(l)$ is the proportion of observations of class c in node l: $f_c(l) = \frac{N_c(l)}{N_0(l)+N_1(l)}$. The measure $I_h \to 1$ when each terminal nodes have about the same number of observations of each class and $I_h \to 0$ when the terminal nodes become pure. The classification analysis was performed using the `sklearn` module [5] version *0.20.1*.

3.2 Propofol TCI statistics during the entire maintenance period

We compare the averaged TCI for groups of patients with no IES vs IES (Supplementary Fig. 8: we find that the no-IES group had a slightly higher TCI during maintenance compared to the IES group (median [IQR)]; TCI=3.7 [3.3, 4] μg ml^{-1} no IES vs 3.2 [3, 3.6] μg ml^{-1} for IES patients). This result could be understood by an adaption of the propofol dose to the patients response. This result confirms that here, propofol TCI cannot be used to predict the appearance of IES.

3.3 αS precedes IES dynamics

To confirm the predictive value of αS, we show here that their statistics preceded the ones of the IES. We first normalized each variable associated to αS events: the IES fraction P_{IES}, αS duration Dur_α, frequency F_α and average amplitude A_α, with their respective plateau value (trace averaged over $[20, 35]$min). For each trace, we then approximated numerically the times t_∞ to reach 99% of the final value. For that purpose, we approximated each curve either with a sigmoidal

$$y(t) = \frac{1}{1 + \exp\left(-4\frac{(t-t_0)}{\tau}\right)}, \tag{2}$$

or with an exponential curve

$$y(t) = 1 - e^{-(t-t_0)/\tau}, \tag{3}$$

where t_0 and τ are constants. We obtained for the sigmoid model (2), (resp. exponential, (3)) that $t_\infty = t_0 + 1.148\tau$ (resp. $t_0 + 4.60\tau$). Fitting was performed in MATLAB R2018a.

We first computed times for the fraction P_{IES} (dashed red) of IES in the EEG signal and αS durations Dur_α (magenta) and we found that Dur_α plateaus sooner ($t_\infty =$ 13.78min) than P_{IES}, which equilibrates at $t_\infty = 19.54$min (Supplementary Fig. 2A). This result confirms that the variable Dur_α anticiapte the appearance of IES.

Similarly, we computed for the frequency F_α (magenta) that the time to plateau was $t_\infty = 7.74$min (Supplementary Fig. 2B). The frequency F_α reached its steady-state before P_{IES} and αS duration Dur_α. Interestingly, as estimated above, the average time of the first IES occurrence was $\langle t_{IES} \rangle = 11.88min$, which thus happens almost 4 minutes after F_α has reached its plateau. Additionally, we remarked that during induction, F_α behavior followed the increase of αS fraction in the EEG signal P_α (yellow). These results show that F_α steady-state is rapidly established during the induction phase and occurs in average before first IES. Finally, we found that the average αS amplitude A_α reached its plateau value at $t_\infty = 18.89$min, which is similar to the one of IES.

To summarize, these results highlight that αS dynamics precedes IES ones, but also that αS variables possess different timescales. In particular, both αS duration Dur_α and

average amplitude A_α had a slow timescale and reached their steady-state value during the maintenance. However, the occurrence frequency F_α equilibrium was rapidly established during the induction phase.

3.4 IES prediction based on δ-power and alpha-to-delta ratio

The depth of anesthesia can be quantified by several markers such as the delta band power or the alpha-to-delta ratio [6]. The performance of these markers in predicting the occurrence of IES during the first 35min, can be evaluated by comparing the αS 'P_α proportion slope a' to the alpha-to-delta ratio or the delta power. For that goal, we estimated these parameters during the induction phase.

We computed the power spectrum during the induction phase for each group, presenting or not IES. We estimated the alpha-to-delta ratio (Supplementary Fig. 6) and found that the power spectrum between the two groups (with and with no IES) was significantly different.

Interestingly, using a univariate logistic model, we show here that αS features ('P_α proportion slope a') outperformed alpha-to-delta ratio by 20% (85% vs 65%, respectively (Supplementary Table 6). Similarly, the δ-power had an accuracy of 73.75% vs 75% and 85% for αS amplitude (A_α) and 'P_α proportion slope a'.

A multivariate analysis shows that the δ-power and the αS amplitude are independent (Supplementary Fig. 7). Yet, using these two variables, we found an accuracy of 76.25% and an AUC of 0.825 which remains much below the performance obtained with the 'P_α proportion slope a' variable alone. Finally, when we compare the δ-power and 'P_α proportion slope a', we found that these variable are not independent: in that case the δ-power was no longer significant (p=0.363), while the 'P_α proportion slope a' is clearly the most predictive variable with a p-value <0.0001. These results show that αS are most suited to predict IES occurrences.

References

[1] Gavin C Cawley and Nicola LC Talbot. On over-fitting in model selection and subsequent selection bias in performance evaluation. *Journal of Machine Learning Research*, 11(Jul):2079–2107, 2010.

[2] Ingrid Daubechies. *Ten lectures on wavelets*, volume 61. Siam, 1992.

[3] Jerome Friedman, Trevor Hastie, and Robert Tibshirani. *The elements of statistical learning*, volume 1. Springer series in statistics New York, NY, USA:, 2001.

[4] Gilles Louppe, Louis Wehenkel, Antonio Sutera, and Pierre Geurts. Understanding variable importances in forests of randomized trees. In *Advances in neural information processing systems*, pages 431–439, 2013.

[5] F. Pedregosa, G. Varoquaux, A. Gramfort, V. Michel, B. Thirion, O. Grisel, M. Blondel, P. Prettenhofer, R. Weiss, V. Dubourg, J. Vanderplas, A. Passos, D. Cournapeau, M. Brucher, M. Perrot, and E. Duchesnay. Scikit-learn: Machine learning in Python. *Journal of Machine Learning Research*, 12:2825–2830, 2011.

[6] PL Purdon, KJ Pavone, O Akeju, AC Smith, AL Sampson, J Lee, DW Zhou, K Solt, and EN Brown. The ageing brain: age-dependent changes in the electroencephalogram during propofol and sevoflurane general anaesthesia. *British journal of anaesthesia*, 115(suppl_1):i46–i57, 2015.
