# Supplementary material for: Alpha rhythm collapse predicts iso-electric suppressions during anesthesia
Source: Commun Biol. 2019 Sep 2;2:327. doi: 10.1038/s42003-019-0575-3 (PMC6718680; doi:10.1038/s42003-019-0575-3)
Supplement: Supplementary file 2 — Description of Additional Supplementary Files [file 42003_2019_575_MOESM2_ESM.docx]

**Descriptions of additional supplementary data**

**I) Description of the supplementary data 1**

The .xlsx file contains three sheets related to figures 1, 2 and 3 respectively, from the main text. The data of a given figure is always preceded by the figure identity in bold (ex. **Fig. 1F**).

The first sheet (Fig. 1) includes the values to draw (boxplot 1F and 1H), as well as the time series to construct fig. 1G. In particular we have included for the fig. 1G the means (thick red and yellow lines), followed by the standard deviations (shaded areas in the figure) such that ‘up yellow’ and ‘yellow bottom’, that refer to upper and lower graphs, respectively in fig. 1G.

In the second sheet, the three first columns correspond to the time series of the signals shown in Fig. 2A. For figs 2B to 2E, we provided data for the time and the variables values for the population with IES, and without IES.

In the third sheet, the first values encountered are the ones shown in the correlation matrix Fig. 3A. The first column of data tagged ‘Fig.3B’ refers to the False Positive Rate (FPR) followed by column associated to the different features analyzed in the main text. A similar organization of the data is used for Fig. 3C. Data in Fig.3D include~~s~~ the average and standard deviation of the importance value for each considered variable. Finally, the three last columns in sheet 3 account for the x-y coordinates and associated group (0 = no-IES, 1=IES) for each point~~s~~ presented in fig. 3E.
